# Use of non-invasive transcutaneous auricular vagus nerve stimulation: neurodevelopmental and sensory follow-up

**DOI:** 10.3389/fnhum.2023.1297325

**Published:** 2023-11-09

**Authors:** Turki Aljuhani, Patricia Coker-Bolt, Lakshmi Katikaneni, Viswanathan Ramakrishnan, Alyssa Brennan, Mark S. George, Bashar W. Badran, Dorothea Jenkins

**Affiliations:** ^1^Division of Health Science and Research, Medical University of South Carolina, Charleston, SC, United States; ^2^Department of Occupational Therapy, College of Applied Medical Sciences, King Saud Bin Abdulaziz University for Health Sciences, Riyadh, Saudi Arabia; ^3^Doctorate of Occupational Therapy Program, Hawai’i Pacific University, Honolulu, HI, United States; ^4^Department of Pediatrics, Medical University of South Carolina, Charleston, SC, United States; ^5^Department of Public Health Sciences, Medical University of South Carolina, Charleston, SC, United States; ^6^Department of Psychiatry and Behavioral Sciences, Medical University of South Carolina, Charleston, SC, United States; ^7^Ralph H. Johnson VA Medical Center, Charleston, SC, Unites States

**Keywords:** taVNS, feeding delay, infants, neurodevelopmental delay, sensory processing

## Abstract

**Objective:**

To assess the impact of non-invasive transcutaneous auricular vagal nerve stimulation (taVNS) paired with oral feeding on long-term neurodevelopmental and sensory outcomes.

**Method:**

We tested 21 of 35 children who as infants were gastrostomy tube (G-tube) candidates and participated in the novel, open-label trial of taVNS paired with oral feeding. To evaluate possible effects on development at 18-months after infant taVNS, we performed the Bayley-III (*n* = 10) and Sensory Profile (SP-2, *n* = 12) assessments before the COVID pandemic, and Cognitive Adaptive Test (CAT), Clinical Linguistics and Auditory Milestone (CLAMS), Ages and Stages Questionnaire (ASQ), and Peabody Developmental Motor Scales-2 gross motor tests as possible during and after the pandemic. We compared outcomes for infants who attained full oral feeds during taVNS (‘responders’) or received G-tubes (‘non-responders’).

**Results:**

At a mean of 19-months, taVNS ‘responders’ showed significantly better general sensory processing on the SP-2 than ‘non-responders’. There were no differences in other test scores, which were similar to published outcomes for infants who required G-tubes.

**Conclusion:**

This is the first report of neurodevelopmental follow-up in infants who received taVNS-paired feeding. They had similar developmental outcomes as historical control infants failing oral feeds who received G-tubes. Our data suggests that infants who attained full oral feeds had better sensory processing.

## 1. Introduction

Feeding difficulties are common for preterm and term infants with brain injury during their stay in the neonatal intensive care unit (Martinez-Biarge et al., 2012; Shandley et al., 2020). Infants who do not attain full oral feeds after term age have limited options except to continue to practice oral feeding with therapists and many ultimately require gastrostomy tube placement (G-tube) (Warren et al., 2019).

Non-invasive transcutaneous auricular vagal nerve stimulation (taVNS) is a novel intervention that may improve oromotor skills and increase neuroplasticity when paired with the motor activity of bottle feeding (Badran et al., 2018). Based on extensive work in animals and human adults after stroke, vagus nerve stimulation (VNS) simultaneously paired with a motor activity has been shown to increase acetylcholine and norepinephrine neurotransmitters important to learning, induce cortical neuroplasticity, and improve motor skills after stroke (Groves and Brown, 2005; Ben-Menachem et al., 2015; Dawson et al., 2016; Kimberley et al., 2018; Dawson et al., 2021). VNS paired with motor rehabilitation is FDA approved in adults after stroke (Dawson et al., 2021). Our group has been at the forefront in developing a non-invasive method of taVNS that activates the afferent vagus nerve network via the auricular branch of the vagus nerve in infants while feeding (Badran et al., 2020). In a first-of-its-kind open-label pilot study in infants who were candidates for G-tube due to failing oral feeding, we demonstrated that taVNS paired with bottle feeding is feasible, safe and may improve oromotor-feeding skills compared to the period before taVNS treatment, leading to an increase in the number of infants who attain full oral feeds (Badran et al., 2020; Jenkins et al., 2023). Furthermore, infants who achieved full oral feeds during taVNS-paired feeding treatment also showed significant gains in white matter integrity (fractional anisotropy) in the superior longitudinal fasciculus and white matter complexity (radial kurtosis) in the corticospinal tract at the cerebellar peduncles and external capsule, indicating aspects of neuroplasticity in the ‘responders’ that were not present in the ‘non-responders who received a G-tube. The change in radial kurtosis per week of development was significantly higher in responders versus non-responders in the right corticospinal tract at the cerebellar peduncles, which includes oromotor tracts, and in the right external capsule, a basal cholinergic fiber route whose activation is consistent with prior reports of vagus nerve stimulation inducing cholinergic responses required for motor learning. The change in FA was greater in responders than non-responders in the right superior longitudinal fasciculus, a large complex tract associated with attention in visuospatial tasks (Janelle et al., 2022). For functional correlation, we also found increased head and neck control from before to after taVNS paired with feeding in the infants who achieved full oral feeds compared with infants who required G-tubes (Aljuhani et al., 2022). The improvements in head and neck control are consistent with prior studies of VNS which shows that functional improvements are task specific (Engineer et al., 2019). In this vulnerable period of perinatal neurodevelopment it is unknown if taVNS in infants affects later neurodevelopment.

Multiple studies have demonstrated that early feeding difficulties may predict later neurodevelopment delays (Tsai et al., 2010; Northam et al., 2012; Jadcherla et al., 2017; Zhang et al., 2017). In this first report of longer-term impact of taVNS-paired feeding, we investigated whether there were neurodevelopmental and sensory effects at 18 months in children who received taVNS treatment as infants. We also compared our outcomes with published data from historical controls of infants who had received G-tubes (Jadcherla et al., 2017), as these infant cohorts have significant global developmental delays at 18 months that may persist up to 5 years (Crapnell et al., 2015; Wolthuis-Stigter et al., 2015, 2017). Infants with feeding difficulties often have disorganized motor and behavioral responses to sensory stimulation in general and show difficulty calming and self-soothing without significant support from staff and caregivers. Studies have demonstrated an association between feeding difficulties in children and atypical sensory processing scores. However, few studies have examined the influence of infants’ feeding difficulties on early sensory processing. Since atypical sensory possessing can be a cause of feeding difficulties (Davis et al., 2013; Yi et al., 2015; Tauman et al., 2017), we hypothesized there may be an early relationship between the infant’s sensory processing and feeding.

The aims of this sub-study were (1) to determine whether infants who were treated with taVNS-paired feeding have similar neurodevelopmental performance and sensory processing scores at 18 months compared with published data on preterm infants who were discharged with G-tubes (Jadcherla et al., 2017), and (2) to determine if there are Neurodevelopmental Impairments (NDI) or sensory differences in infants who attained full oral feeds after taVNS treatment (responders) versus infants treated with taVNS who required a G-tube (non-responders). Before the COVID pandemic, we administered the Bayley III in the home or clinic setting to assess motor, cognitive and language skills, and the Toddler SP-2 to evaluate sensory processing abilities. After the COVID pandemic, infants completed follow-up at a high-risk clinic using a standard clinic battery of developmental assessments. We postulated that Bayley III/ developmental scores would be similar across our cohort of infants compared with historical controls, and that the ‘responders’ who achieved full oral feeds would have more typical motor and sensory profiles than would ‘non-responders’.

## 2. Materials and methods

### 2.1. Design

This is a prospective cohort study of at-risk infants enrolled into a taVNS feeding study registered in Clinicaltrials.gov (NCT04643808) and approved by the Institutional Review Board at the Medical University of South Carolina (MUSC Pro #67997), who has ruled the taVNS device a non-significant risk device. Parental consent was obtained prior to enrollment. The primary study recruited 35 infants from 2017 to 2022 who had failed oral feeding by term age equivalent and were referred for G-tube placement in this open-label trial of taVNS paired with once or twice daily bottle feedings for 2 weeks (Badran et al., 2020; Jenkins et al., 2023).

taVNS -paired feeding protocol was implemented as previously described (Badran et al., 2020; Jenkins et al., 2023): Ear electrodes were placed at the outer and inner tragus during the feeding, and taVNS was delivered by an electronic pulse generator (Digitimer, DS7AH, Digitimer LTD; or Soterix taVNS EPG, Soterix Medical Inc) using 25 Hz, 500 μsec, square wave pulse of microcurrent at 0.1 mA less than perceptual threshold determined by infant response to stimulation with a facial change or leg extension. Stimulation was switched on during active sucking and swallowing and off during rest or burping, during one or two 30 min bottle feedings daily. The treatment period was 2 weeks, with 1–2 week extension if the infant was making progress with daily oral feeding volumes, but not quite at full oral feeds.

**Figure 1:**
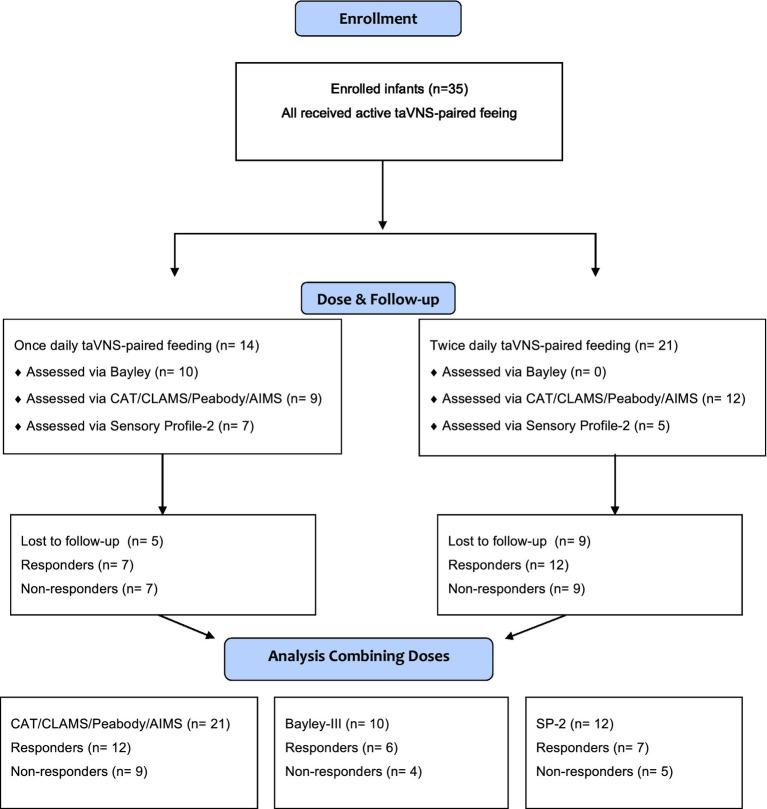
Flow chart of all enrolled participants of the study.

Developmental testing: The study continued to enroll during the COVID pandemic, and the Specific Test Early infant Performance STEP assessments were performed before and after the taVNS treatment period while infants were in the hospital (Gower et al., 2019). All other developmental assessments were completed at either the parent’s home or an MUSC outpatient clinic, depending on the families’ preference, from 2019–2023. Developmental follow-up at 18 months corrected age (CA) was limited by the requirement for parents to bring infants back to hospital and with some parents refusing to allow investigators in their homes to perform Bayley developmental assessments during the time of the pandemic. High-risk clinic visits with data collection of standard, validated developmental assessments resumed after the pandemic (Figure 1).

### 2.2. Outcome measures

We performed 18-month follow up assessments on 10 of these children during the COVID pandemic using the Bayley-III assessment. A total of 21 children were later seen at the high-risk infant clinic and were assessed by the Cognitive Adaptive Test (CAT), Clinical Linguistic and Auditory Milestone Scale (CLAMS), Modified Peabody Developmental Gross Motor Scale, Modified Checklist for Autism in Toddlers (M-CHAT), and Ages and Stages Questionnaire (ASQ) (Supplementary Table S1 online). Twelve children’s parents completed the Sensory Profile-2 caregiver questionnaire (SP-2).

### 2.3. Data analysis

In these exploratory analyses we compared differences between the responders and non-responders on the Bayley-III and SP-2 scores via ANCOVA, controlling for gestational age (GA) at birth. We used a Pearson correlation to determine relationships between short and long-term neurodevelopmental tests (STEP, Bayley-III, SP-2), the independent sample t-test for differences between responders and non-responders in CAT/CLAMS, and Fisher’s exact test for differences in number of participants who scored typical/atypical on the SP-2 (SPSS V.27.0; SPSS Inc., Chicago, IL).

## 3. Results

### 3.1. Participants

Out of the 21 children who attended the high-risk clinic with complete follow-up data, 12 were responders and 9 were non-responders. The mean age at follow-up was 20.5 ± 9 months (*n* = 21) (Table 1). There were no significant differences between the groups’ characteristics for age at the time of starting taVNS intervention, weight, height, occipital frontal circumference, and ADI. Infants born prematurely in this cohort (*n* = 16) had been attempting po for mean of 47 ± 20 days (range 22–107 days) prior to taVNS treatment, while the 5 near-term and term HIE infants attempted po for a mean 30 ± 8 days. Responders reached full po feeds in a mean of 10 ± 7 days. CNS injuries were common in these cohorts (Table 1).

**Table 1:** Demographic information on infants attended the high-risk clinic.

			taVNS subjects (*n* = 21)	Responders (Full PO, *n* = 12)	Non-responders (G-tube, *n* = 9)
Sex	Male		12	7	5
	Female		9	5	4
GA at birth (weeks)			30.2 ± 4.6	29.7 ± 4.7	30.8 ± 4.6
GA at start of taVNS (weeks)			44.2 ± 4.2	44.3 ± 4.8	44.1 ± 3.7
Days attempting po prior to taVNS (days)			43 ± 19	47 ± 25	37 ± 7
Age at follow-up (months)			20.5 ± 9.0 (9.6–37.6)	21.3 ± 9.0 (9.6–37.6)	19.6 ± 9.6 (9.7–31.1)
Intraventricular hemorrhage (IVH)	Grade I		8	6	2
	Grade II		1	0	1
	Grade III		1	1	0
	Grade IV		1	0	1
Hypoxic ischemic encephalopathy (HIE)			5	2	3
Periventricular leukomalacia (PVL)			4	1	3
Sepsis			5	1	4
Necrotizing enterocolitis			3	3	0
Area deprivation index			4.5 ± 2.7	4.8 ± 2.8	4.0 ± 2.0
Specific test of early infant motor performance (pre taVNS)			14.6 ± 4.8	14.3 ± 6.2	14.9 ± 3.7
Specific test of early infant motor performance (post taVNS)			16.1 ± 3.5	16.4 ± 3.7	15.7 ± 3.5
Cognitive adaptive test (CAT)	Mean, SD		88 ± 25	87 ± 30	89 ± 18
	Category	Average (*n*, %)	15 (71)	7 (58)	8 (89)
		Delayed	1 (5)	1 (8)	0
		Very delayed	5 (24)	4 (33)	1 (11)
Clinical linguistic and auditory milestone scale (CLAMS)	Mean, SD		87 ± 25	88 ± 31	86 ± 18
	Category	Average	12 (57)	7 (58)	5 (56)
		Delayed	3 (14)	0	3 (33)
		Very delayed	6 (29)	5 (42)	1(11)
Modified peabody developmental gross motor scale (PDMS-2)	Mean, SD		89 ± 20	94 ± 19	83 ± 22
Ages and stages questionnaire (ASQ): gross motor	Pass		12 (57)	6 (50)	6 (67)
	Fail		6 (28)	4 (33)	2 (22)
	At risk		3 (14)	2 (17)	1 (11)
ASQ: fine motor	Pass		12 (57)	7 (58)	5 (56)
	Fail		6 (28)	4 (33)	2 (22)
	At risk		3 (14)	1 (8)	2 (22)
ASQ: communication	Pass		12 (57)	7 (58)	5 (56)
	Fail		5 (24)	3 (25)	2 (22)
	At risk		4 (19)	2 (17)	2 (22)
ASQ: problem solving	Pass		13 (62)	6 (50)	7 (78)
	Fail		5 (23)	3 (25)	2 (22)
	At risk		3 (14)	3 (25)	0
ASQ: personal-social	Pass		8 (38)	5 (42)	3 (33)
	Fail		9 (43)	5 (42)	4 (44)
	At risk		4 (19)	2 (17)	2 (22)
Modified checklist for autism in toddlers (M-CHAT, *n* = 14)	Pass		12 (86)	7 (88)	5 (83)
	Fail		1 (7)	1 (12)	0
	At risk		1 (7)	0	1 (17)

Mean (SD) or number (%).

*p-values for data points in Supplementary Table S3.1 are all > 0.05.

Performance categories: very delayed (>2SD); delayed (1 to 2SD); average (±1SD).

A total of 10 children at mean of 19 ± 1.8 months of age participated in testing cognitive, language, and motor skills via the Bayley-III. There were no significant differences between the responders (*n* = 6) and non-responders (*n* = 4) groups in age at the follow-up assessment, CNS abnormalities, birth information, medical history, or taVNS information with this limited sample size (Supplementary Table S3 online).

The parents of 12 children (7 responders, 5 non-responders) completed all sections of the SP-2 caregiver questionnaire (Table 2). There were no significant differences in demographic characteristics or days attempting po feeds prior to taVNS treatment between groups (*p* = 0.25), although one responder had attempted oral feeding for 107 days prior to enrollment in the taVNS study.

**Table 2:** Demographics information participants (*n* = 12) who completed the 18-month follow-up SP-2 questionnaire, divided into responders vs. non-responders.

	Total *N* = 12	Responders (Full PO feeds) *N* = 7	Non-responders (G-tube) *N* = 5	*p*-value*
Birth information				
Male	7	4	3	
Female	5	3	2	
GA at birth (weeks)	30.4 ± 4.7	32.1 ± 5.1	27.9 ± 3.0	0.14
Birth weight (grams)	1706.7 ± 1252.6	1955 ± 1335	1359.0 ± 1175.6	0.44
Medical history				
Clinical sepsis	5	2	3	0.31
Persistent pulmonary hypertension of the newborn (PPHN)	5	3	2	0.69
Patent ductus arteriosus (PDA)	3	3	0	0.16
Intraventricular hemorrhage (IVH)	8	Grade I [1] Grade II [1] Grade III [1]	Grade I [3] Grade II [2]	0.25
Hypoxic ischemic encephalopathy (HIE)	6	4	2	0.5
Periventricular leukomalacia (PVL)	2	1	1	0.68
taVNS information (Mean ± SD)				
GA at taVNS start (weeks)	40.2 ± 2.8	41.8 ± 1.8	42.8 ± 4.0	0.54
Days attempting PO prior to taVNS	37.25 ± 18.2	28.4 ± 15	49.6 ± 15.5	0.04
Total number of taVNS sessions	21.1 ± 12.5	16.6 ± 7.4	27.4 ± 16.3	0.15
Specific test of early infant motor performance (pre taVNS)	13.5 ± 4.6	13.8 ± 5.2	13.2 ± 3.6	0.8
Specific test of early infant motor performance (post taVNS)	14.2 ± 3.4	14.8 ± 4.1	14.6 ± 3.9	0.7
Age at follow-up assessment (months)	19.0 ± 1.4	19.0 ± 1.6	18.9 ± 1.1	0.44

Independent *t*-test or Fisher’s exact test, PO = oral feed.

### 3.2. High risk clinic assessments results

#### 3.2.1. Cognitive adaptive test and clinical linguistic and auditory milestone scale

For the larger cohort assessed in high-risk clinic, there were no significant differences in the mean CAT or CLAMS scores or in the number of infants in the delayed categories between the responders (*n* = 12) and non-responders (*n* = 9) (Table 1).

#### 3.2.2. Modified peabody developmental gross motor scale

The mean score of the Gross Motor Scale of the Modified Peabody and number of infants in the Delayed/ Very Delayed category were also similar between the responders and non-responders (Table 1).

#### 3.2.3. Ages and stages questionnaire

All domains of the ASQ (communication, gross motor, fine motor, problem solving, and personal-social) were completed during the high-risk clinic visit. There were no significant differences between the two groups on any domain of the ASQ. Nine of the 21 children failed the ASQ including five responders and four non-responders. The most common domain that children failed was the ASQ personal social domain (Table 1).

#### 3.2.4. Area of deprivation index

The mean ADI for the group was 4.5 ± 2.6. The mean for the responders (*n* = 11) was 4.8 ± 2.9, and for the non-responders (*n* = 9) 4.0 ± 2.4, which were not significantly different (Table 1).

#### 3.2.5. Modified checklist for autism in toddlers

Fourteen out of the 21 children had M-CHAT scores. Only one child failed the M-CHAT and was referred for testing, indicating increased risk for an autism diagnosis (Table 1).

### 3.3. Bayley-III results

Bayley-III results were very similar between our cohort (*n* = 10) and published data on preterm infants who were discharged with a G-tube (Table 3) (Jadcherla et al., 2017; Warren et al., 2019). There were no significant differences between responders and non-responders across cognition, receptive language, fine motor and gross motor domains (*p* > 0.5). Infants who responded and achieved full feeds during taVNS had slightly higher mean Bayley-III scaled scores than non-responders, in cognition (+1.6), receptive language (+0.3), fine motor (+0.7), and gross motor (+1.6), but lower expressive language score (−1.7).

**Table 3:** A comparison of Bayley-III scores between Jadcherla et al. (2017) study and our cohort results.

	All taVNS infants (*n* = 10)	taVNS responders (Full po, *n* = 6)^a^	taVNS Non-responders (G-tube, *n* = 4)^a^	*p*-value^†^	Jadcherla (Full-PO *n* = 177)^b^	Jadcherla (G-tube *n* = 77)^b^	*p*-value*
Age (mo)	19.44 ± 1.8	19.95 ± 2.5	18.7 ± 0.3	0.6	18.4 ± 1.8	18.3 ± 1.3	0.8
Cognitive composite score	90 (24)	92 (25)	80 (25.5)	0.9	90 (20)	80 (20)	<0.01
Receptive scaled score	8 (5.5)	10 (5)	7 (5.5)	0.5	8	6 (4)	0.01
Expressive scaled score	5.5 (4)	5 (1)	7 (8)	0.8	7 (4)	6 (4)	0.06
Fine motor scaled score	6 (8)	8 (8)	5.5 (6)	0.5	9 (4)	7 (5)	0.01
Gross motor scaled score	6.5 (4)	9 (4)	5.5 (6.5)	0.5	8	5 (5)	<0.01

^a^Value stated as mean (standard deviation) data is normally distributed.

^b^Value stated as median (IQR).

^*^p-value between Full-po and G-tube infants as reported in Jadcherla et al. (2017).

^†^ANCOVA test controlling for GA.

### 3.4. Toddler SP-2 caregiver questionnaire results

The results of the SP-2 are presented in two sections: sensory behaviors and sensory quadrants. The scores on the seven sensory behaviors (general, auditory, visual, touch, movement, oral, and behavior) are presented in (Table 4). The general sensory behavioral scores showed a statistically significantly difference between the responders and non-responders, with higher (more atypical, > +1SD) sensory scores in the non-responder group (*p* = 0.04). Moreover, nearly all children in the non-responder group (4/5, 80%) had atypical sensory scores compared with only 1 out of 7 (14%) in the responder group (*p* = 0.03). These data indicate that the non-responder infants were more responsive (overly sensitive) than responder infants to overall sensory information, which may impact their ability to interact in age-appropriate activities requiring processing of sensory information. There were no differences in mean oral sensory scores between infants who attained full oral feeds and those that failed and required G-tube. Both groups had relatively high, but still typical oral sensory scores (responders = 13.1, non-responders = 13.8; normal range 6–15) (Dunn, 2014).

**Table 4:** Results of the SP-2 sensory behavior section using ANCOVA between the responders and non-responders.

Sensory behavior (typical range)	Responders (full feed) *n* = 7		Non-responders (G-tube) *n* = 5		*p*-value
	Mean (SD)	Atypical score (*n*)	Mean (SD)	Atypical score (n)	
General [11-22]	16.8 (5.1)	1	**24.0** (3.5)	4	0.04*
Auditory [6-14]	10.1 (2.8)	1	**15.0** (5.7)	2	0.15
Visual [11-19]	16.6 (3.9)	1	17.6 (4.7)	2	0.96
Touch [6-13]	8.1 (1.9)	0	**14.0** (9.2)	1	0.18
Movement [13-20]	16.4 (3.4)	1	18.0 (4.2)	1	0.57
Oral [6-15]	13.1 (5.9)	2	13.8 (3.8)	1	0.80
Behavior [7-14]	9.6 (1.5)	0	15.2 (8.3)	1	0.20
Quadrants (typical range)					
Seeking [23-33]	28.7 (4.9)	1	31.2 (2.9)	2	0.55
Avoiding [11-21]	16.6 (4.9)	1	**22.0** (8.7)	2	0.20
Sensitivity [13-27]	21.6 (3.9)	1	**27.8** (7.5)	2	0.14
Registration [10-21]	17.4 (7.5)	1	**21.8** (10.7)	2	0.51

Higher or lower score than typical (in bold) indicates atypical sensory behavior.

The second SP-2 section presents the results from the seeking, avoiding, sensitivity, and registration quadrants (Table 4). These scores reflect that the child is responding to some sensory stimuli much more than others in specific SP-2 quadrants: one toddler scored atypical (+2SD) in the Avoidance quadrant, two children in the Sensitivity quadrant, and one child in the Poor Registration quadrant. While the mean scores of these sensory quadrants were more atypical (higher) in the non-responder group than in the responder group, there were no statistically significances in any quadrant.

### 3.5. STEP and Bayley-III

There was a significant positive correlation between pre-taVNS STEP scores performed at term age equivalent up to 3 months CA and Bayley-III scores at 18 months in cognitive (*r* = 0.75; *p* = 0.03), fine motor (*r* = 0.75; *p* = 0.03), and gross motor domains (*r* = 0.75; *p* = 0.03, *n* = 10). However, pre-taVNS STEP scores did not correlate with the Bayley-III language domain scores.

### 3.6. Bayley-III and toddler SP-2 caregiver questionnaire results

The Pearson correlation showed a significant negative association between the Bayley-III motor composite score (fine and gross scores) and the oral sensory performance (*r* = −0.80; *p* = 0.03, *n* = 7), and between the fine motor scaled sore and oral sensory performance (*r* = − 0.82; *p* = 0.02). This indicates that lower Bayley-III motor scores are associated with more atypical oral sensory issues in this cohort.

## 4. Discussion

This is the first study to investigate the long-term neurodevelopmental and sensory outcomes of children who as infants received taVNS paired with bottle feeding. The results of data from our cohort show comparable long-term neurodevelopmental outcomes in cognitive, receptive language, fine and gross motor skills, and sensory performance at 18–20 months as published data from preterm infants who received G-tube placement in early infancy (Jadcherla et al., 2017; Warren et al., 2019). This follow-up data provides support that early, non-invasive brain stimulation via taVNS-paired with feeding in infants does not have a negative impact on overall development at a mean of 20 months. Though sample size is small, assessment of sensory processing abilities showed that infants who achieved full oral feeds prior to hospital discharge (responders) had more typical (normal) general sensory processing patterns comparison to infants who received a G-tube (non-responders). Differences in Bayley III scores and other sensory performance scores did not reach the level of significance, but gross and fine motor scores correlated with oral sensory performance on the SP-2.

Early feeding failure represents the first delay in attaining a significant developmental milestone in infancy and has been used as a short-term neurologic outcome in a study of HIE infants (Onda et al., 2022). Our cohort was clearly delayed in their oromotor skills and feeding abilities, and the majority had CNS injury evident on neuroimaging studies. There was a discrepancy in the number of infants with cystic PVL (more in the non-responder group), which is the presence of cystic areas from necrosis, which resolve over weeks with atrophy, gliosis or resorption/repair. However, the vast majority of white matter injury is non-cystic in nature and not easily detected by routine ultrasound or qualitative MR imaging. Conditions associated with neuroinflammatory injury, such as necrotizing enterocolitis and sepsis, were similar between groups as a whole.

We enrolled term equivalent or older infants who had been referred for a G-tube placement after these infants attempted to oral feed for an average of 43 days. Data from other studies suggest that the infants in our study were at higher risk of later neurodevelopmental impairments than preterm infants who attained full oral feeds prior to hospital discharge (Martinez-Biarge et al., 2012; Wolthuis-Stigter et al., 2017). Greater NDI has been reported from the large cohorts of extremely low birth weight infants who received G-tubes in the Neonatal Research Network report (*n* = 333, 61% NDI) and in infants <37 weeks GA at birth referred to a neonatal feeding program (*n* = 77 infants with G-tube, 50% motor and 45% cognitive delays) (Jadcherla et al., 2017; Warren et al., 2019).

We observed significant associations between the Bayley-III motor composite score (fine and gross scores) and Sensory Profile-2 results. Lower composite scores for gross and fine motor skills in infants receiving taVNS were related to more atypical oral sensory processing. Other studies also found a relationship between atypical SP-2 sensory processing scores and delays in Bayley III motor, cognition, and language domains (Eeles et al., 2013; Yi et al., 2015). For example, more typical scores for auditory, touch, and oral sensory processing have been associated with higher language composite scores, while typical scores for touch and movement processing have been correlated with higher cognitive composite scores on the Bayley-III. Atypical sensory registration and avoidance quadrants on the SP-2 have been associated with lower motor composite scores on the Bayley-III (Eeles et al., 2013).

In a limited cohort of 12 infants, 20-month follow-up scores on the SP-2 revealed that significantly more infants in the taVNS responder group had typical sensory processing scores compared to the non-responders who did not achieve full oral feeds. This suggests that infants who achieved full oral feeds during the taVNS paired feeding protocol, were more likely to react appropriately to typical everyday sensory stimuli in the environment than infants who required G-tube placement prior to discharge. Although not statistically significant between responders and non-responders in this small sample with variability, mean scores in the auditory, touch, behavior, and avoiding, sensitivity, registration quadrants of the SP-2 in the non-responders group were atypical (>1SD and > 2SD from the mean for their age), in the non-responders group. These findings are limited by sample size constraints, but will help direct future studies of sensory development in children who have difficulty mastering oromotor skills as infants.

The vagus nerve plays a key role in the interface between the higher central nervous system circuits and autonomic control circuitry of the brain stem (Hulsey et al., 2016). Mechanisms of VNS induction of synaptic plasticity involve an increase in norepinephrine in the locus coeruleus involved with attentional motor learning, and an increase in the acetylcholine neurotransmission in the basal forebrain projections to the thalami and cortical areas (Elger et al., 2000; Chae et al., 2003). VNS has been shown to improve motor learning after stroke by inducing cortical plasticity while requiring acetylcholine activation for circuit modulation and motor learning (Engineer et al., 2015; Bowles et al., 2022). taVNS activates similar brain regions as VNS in fMRI studies(Dietrich et al., 2008; Badran et al., 2018; Briand et al., 2020). Thus, while there are well-established mechanisms of VNS-induced plasticity that may explain our preliminary data of taVNS’ effect on feeding skills (Porter et al., 2012; Engineer et al., 2015; Badran et al., 2023), we do not yet know whether the responders’ typical sensory profile scores are associated with taVNS treatment or latent behavioral proclivities due to less CNS injury or dysmaturity. Further, po feeding and the interactions engendered with the parent or care providers, may be responsible for the more typical sensory performance in the group that reached full oral feeds.

While pairing taVNS with a motor activity seems to boost activity dependent neuroplasticity in adults after stroke (Redgrave et al., 2018), and in the infants in this study (Jenkins et al., 2023), little is known about the long-term effects and safety of vagus nerve stimulation in infants. Only one other study has investigated the use of brain stimulation in infants, and that involved a single Transcranial Magnetic Stimulation (TMS) pulse in infants less than 1 year of age (Nemanich et al., 2019). The TMS study was not an intervention study and did not report on the long-term neurodevelopmental outcomes. Thus, although our sample size was limited in this first open-label study, we feel it is important to report this safety data on longer-term neurodevelopmental outcomes in infants treated with taVNS paired with bottle feeding.

There were differences in this cohort that returned for developmental testing, and the larger cohort of 35 infants treated with taVNS-paired feeding. CNS injuries, PVL, and HIE were similar in the full cohort. PVL, a cystic WM injury from macroscopic necrosis strongly associated with motor impairments, represents a small portion of WM injury and necrosis, which is largely non-cystic (Van Camp and Steyaert, 2016). PVL was present in 33% of the non-responders group and only 8% of responders in the developmental follow-up cohort. Both sepsis and necrotizing enterocolitis cause neuroinflammation and are associated with white matter injury, and as a group, were equally represented in the reported cohort. Limitations to this study include a small sample size, lack of a concurrent control group and high rate of lost-to follow-up (14/35), largely due to COVID pandemic and restricted access to families during our funding period. Although we were not able to complete Bayley follow-up assessments on our entire cohort due to COVID restrictions, we were able to collect a wide range of neurodevelopmental assessment data in developmental follow-up clinic and felt it was important to report on the outcomes that we were able to obtain. The historical comparison group was largely preterm infants similar to our cohort. Other limitations include that infants received different early interventions after discharge based on attendance for clinic assessments and demonstrated delays that may have impacted long-term follow up results.

## 5. Conclusion

Our results suggest that infants who were G-tube candidates and were treated with taVNS paired oral feeding training had no adverse long-term impacts of the early brain stimulation. Infants who responded during the intervention and who attained full oral feeds had significantly better general sensory processing. Infants in the non-responder group who received a G-tube had similar long-term developmental outcome at 18-months as preterm infants who received G-tubes for feeding failure (Jadcherla et al., 2017). Even with our small sample size, our study provides some assurance that pairing non-invasive taVNS with oromotor training sessions for one to two 30-min daily sessions for two to three weeks early in infancy does not worsen performance on neurodevelopmental tests at 18 months. Future randomized trials of taVNS paired with infant feeding should continue to investigate the long-term impact of taVNS on neurodevelopment.

## Data availability statement

The raw data supporting the conclusions of this article will be made available by the authors, without undue reservation.

## Ethics statement

The studies involving humans were approved by the Institutional Review Board at the Medical University of South Carolina. The studies were conducted in accordance with the local legislation and institutional requirements. Written informed consent for participation in this study was provided by the participants’ legal guardians/next of kin. Written informed consent was obtained from the individual(s), and minor(s)’ legal guardian/next of kin, for the publication of any potentially identifiable images or data included in this article.

## Author contributions

TA: Data curation, Formal analysis, Methodology, Writing – original draft, Writing – review & editing. PC-B: Conceptualization, Formal analysis, Investigation, Methodology, Resources, Supervision, Writing – original draft, Writing – review & editing. LK: Data curation, Methodology, Writing – original draft, Writing – review & editing. VR: Formal analysis, Methodology, Writing – original draft, Writing – review & editing. AB: Formal analysis, Methodology, Writing – original draft, Writing – review & editing. MG: Conceptualization, Resources, Supervision, Validation, Visualization, Writing – original draft, Writing – review & editing. BB: Conceptualization, Funding acquisition, Investigation, Project administration, Resources, Software, Supervision, Validation, Writing – original draft, Writing – review & editing. DJ: Conceptualization, Data curation, Funding acquisition, Investigation, Methodology, Project administration, Resources, Supervision, Validation, Visualization, Writing – original draft, Writing – review & editing.
