# Supplementary material for: Use of non-invasive transcutaneous auricular vagus nerve stimulation: neurodevelopmental and sensory follow-up
Source: Front Hum Neurosci. 2023 Nov 9;17:1297325. doi: 10.3389/fnhum.2023.1297325 (PMC10666166; doi:10.3389/fnhum.2023.1297325)
Supplement: Supplementary file 1 [file Data_Sheet_1.pdf]

Supplementary Material

1 Supplementary Figures and Tables

Table 1 [online]. Outcomes assessment measures used in the study

Assessment	Age range	Purpose	Description and testing time	Format/scoring
**In-patient assessment:**				
Specific Test of Early infant Motor Performance (1)	Birth to 4 months	To assess the quality of early infant movements to identity infants at-risk for later developmental delays	10 items including anti-gravity flexion and extension of the head and neck, movement in the arms and legs, and tone in the shoulder girdle and pelvis. Time: 10 minutes novice, 5 minutes expert	10 items scored on 4-point rating scaled. Established cut-off scores for at-risk category: term ≤16, and 3 months ≤ 22.
**High-risk clinic assessments:**				
Cognitive Adaptive Test (CAT)/ Clinical Linguistic and Auditory Milestone Scale (CLAMS) (2-4)	1 to 36 months	To assess the cognitive, problem-solving and language development of infants and toddlers/	Assessments can be completed by parental report or observation of the child.<br>Time: 20-30 minutes	Both tests: 100-items, developmental quotient normed to 100±15, Significant correlation with the Mental Developmental Index-MDI of the Bayley at 12 and 30 months of age.

Modified Peabody Developmental Gross Motor Scale (5)	Birth to 5 years	To assess aspects of gross motor development including sub-test for stationary, locomotion, and object manipulation.	Semi-structured play with standard administration of specific items based on the child's age. Time: 10-20 minutes	Norm-referenced, with raw score converted to Standard scores, age equivalents, and percentiles. Standard scores 8 -12 are average for age.
Modified Checklist for Autism in Toddlers (M-CHAT) (6, 7)	16 to 30 months	To screen toddlers for Autism Spectrum Disorder	Parent reported questions followed by structured observation. Time: 5-10 minutes	40-items. Scores are divided into risk categories: low, high and medium risk.
Ages and Stages Questionnaire (ASQ) (8-10)	1 to 66 months	To identify infants and children at risk for early developmental delays	Parent questionnaire on development in 5 domains: communication, gross motor, fine motor, problem solving and personal-social. Time: 10-15 minutes	30-items. Established cut-off scores for each domain determine categories of pass, fail, or at risk. A score of 75 in any domain indicates risk for developmental delay.
Area of Deprivation Index (ADI) (11-13)	All ages	To assess the impact of the socioeconomic status on the infant and child development	Standardized index that includes the household, education, employment, housing quality, and income measures taken from the US census and	Scores ranges from 1 (least disadvantage) to 10 (greatest disadvantage) at state level: percentiles of disadvantage at national level.

			American Community Survey data	
**Follow up assessments:**				
Bayley-III (14-18)	1 to 42 months	To evaluate an infant and child’s overall development.	Standardized assessment of cognitive, receptive and expressive language, and fine and gross motor.<br>Time: 40-60 minutes	Norm referenced with raw scores converted to Scaled Scores. Any domain score <9 is > 1 SD below average.
Toddler Sensory Profile-2 caregiver questionnaire(19-21)	7 to 35 months	To measure a child’s sensory processing abilities	Parent questionnaire measure sensory behaviors in 6 domains: general, auditory, visual, tactile, vestibular, and oral Quadrant scores measure seeking, avoiding, sensitivity, and registration.<br>Time: 5-10 minutes	54-items. Domain scores: five-point scale from 1 (almost always) to 5 (almost never). Sensory Quadrant scores: raw scores are age normed and categorized “much less than others” (<2 SD below the mean), “less than others” (1-2 SD below the mean), “just like the majority of others” (<1 SD), “more than others” (1-2 SD above the mean), and “much more than others”(> 2 SD above the

mean)

Table 3 [online]: Demographic information participants (n=10) who completed the 18-month follow-up Bayley III, responders vs. non-responders.

	Total N=10	Responders (Full PO feeds) N=6	Non-responders (G-tube) N=4	*P*-value*
**Birth Information**				
Male	4	2	2	
Female	6	4	2	
Medicaid	5	3	2	0.74
GA at birth (weeks)	29.17 ± 4.2	29.81 ± 5.3	28.21 ± 1.8	0.58
Birth weight (grams)	1283 ± 953.4	1495 ± 1072.9	965 ± 204.5	0.37
**Medical History**				
Clinical sepsis	7	4	3	0.66
Persistent Pulmonary Hypertension of the Newborn (PPHN)	4	3	1	0.45
Patent Ductus Arteriosus (PDA)	3	3	0	0.16
Intraventricular Hemorrhage (IVH)	6	Grade I [1]<br>Grade II [1]<br>Grade III [1]	Grade I [2]<br>Grade II [1]	0.45
Hypoxic Ischemic Encephalopathy (HIE)	4	2	2	0.66

Periventricular Leukomalacia (PVL)	1	0	1	0.54
**taVNS Information (Mean** ± SD)				
GA at taVNS start, weeks	44.2 ± 4.6	45.5 ± 6.4	44.5 ± 3.5	0.79
Days attempting PO prior to taVNS	50 ± 28	50.5 ± 33.7	53.25 ± 16.2	0.88
Total number of taVNS sessions	15.6 ± 4.5	16.3 ± 6.7	17 ± 3.4	0.86
Specific Test of Early Infant Motor Performance (pre taVNS)	14.1 ± 6.3	15.0 ± 7.5	14.7 ± 4.1	0.6
Specific Test of Early Infant Motor Performance (Post taVNS)	14.3 ± 4.3	12.6 ± 4.7	13.5 ± 6.4	0.7
Age at follow-up assessment (months)	19.44 ± 1.8	19.95 ± 2.2	18.7 ± 0.3	0.31

Value stated as Mean (SD)
* Independent t-test and Fisher's Exact test, PO = oral feed

References:

1. Gower L, Jenkins D, Fraser JL, Ramakrishnan V, Coker-Bolt P. Early developmental assessment with a short screening test, the STEP, predicts one-year outcomes. Journal of perinatology : official journal of the California Perinatal Association. 2019;39(2):184-92.

2. Wachtel RC, Shapiro BK, Palmer FB, Allen MC, Capute AJ. CAT/CLAMS. A tool for the pediatric evaluation of infants and young children with developmental delay. Clinical Adaptive Test/Clinical Linguistic and Auditory Milestone Scale. Clinical pediatrics. 1994;33(7):410-5.

3. Voigt RG, Brown FR, 3rd, Fraley JK, Llorente AM, Rozelle J, Turcich M, et al. Concurrent and predictive validity of the cognitive adaptive test/clinical linguistic and auditory milestone scale (CAT/CLAMS) and the Mental Developmental Index of the Bayley Scales of Infant Development. Clinical pediatrics. 2003;42(5):427-32.

4. Hoon AH, Jr., Pulsifer MB, Gopalan R, Palmer FB, Capute AJ. Clinical Adaptive Test/Clinical Linguistic Auditory Milestone Scale in early cognitive assessment. The Journal of pediatrics. 1993;123(1):S1-8.

5. Ramey SL, DeLuca S, Stevenson RD, Case-Smith J, Darragh A, Conaway M. Children with Hemiparesis Arm and Movement Project (CHAMP): protocol for a multisite comparative efficacy trial of paediatric constraint-induced movement therapy (CIMT) testing effects of dosage and type of constraint for children with hemiparetic cerebral palsy. BMJ open. 2019;9(1):e023285.

6. Identifying infants and young children with developmental disorders in the medical home: an algorithm for developmental surveillance and screening. Pediatrics. 2006;118(1):405-20.

7. Robins DL, Casagrande K, Barton M, Chen CM, Dumont-Mathieu T, Fein D. Validation of the modified checklist for Autism in toddlers, revised with follow-up (M-CHAT-R/F). Pediatrics. 2014;133(1):37-45.

8. Squires J. Parent-Completed Developmental Questionnaires: A Low-Cost Strategy for Child-Find and Screening. Infants & Young Children. 1996;9(1).

9. Squires J, Bricker D, Potter L. Revision of a parent-completed development screening tool: Ages and Stages Questionnaires. Journal of pediatric psychology. 1997;22(3):313-28.

10. Squires J, Twombly E, Bricker DD, Potter LW. ASQ-3 User's Guide: Paul H. Brookes Pub.; 2009.

11. Kind AJ, Jencks S, Brock J, Yu M, Bartels C, Ehlenbach W, et al. Neighborhood socioeconomic disadvantage and 30-day rehospitalization: a retrospective cohort study. Annals of internal medicine. 2014;161(11):765-74.

12. Lantos PM, Maradiaga-Panayotti G, Barber X, Raynor E, Tucci D, Hoffman K, et al. Geographic and Racial Disparities in Infant Hearing Loss. Otolaryngology--head and neck surgery : official journal of American Academy of Otolaryngology-Head and Neck Surgery. 2018:194599818803305.

13. Hooper A, Hustedt JT, Slicker G, Hallam RA, Gaviria-Loaiza J, Vu JA, et al. Area Deprivation Index as a predictor of economic risk and social and neighborhood perceptions among families enrolled in Early Head Start. Children and Youth Services Review. 2022;137:106468.

14. Bayley N, Infant S. Bayley scales of infant and toddler development–Third Edition: Technical manual. 2006.

15. Griffiths A, Toovey R, Morgan PE, Spittle AJ. Psychometric properties of gross motor assessment tools for children: a systematic review. BMJ open. 2018;8(10):e021734.

16. Anderson PJ, Burnett A. Assessing developmental delay in early childhood—concerns with the Bayley-III scales. The Clinical Neuropsychologist. 2017;31(2):371-81.

17. Visser L, Ruiter SA, Van der Meulen BF, Ruijssenaars WA, Timmerman ME. Low verbal assessment with the Bayley-III. Research in developmental disabilities. 2015;36:230-43.

18. Duncan AF, Bann C, Boatman C, Hintz SR, Vaucher YE, Vohr BR, et al. Do currently recommended Bayley-III cutoffs overestimate motor impairment in infants born< 27 weeks gestation? Journal of Perinatology. 2015;35(7):516-21.

19. Dunn W. Sensory profile 2 : user's manual. Bloomington, MN.: Psych Corp.; 2014.

20. Dunn W, Brown C. Factor analysis on the Sensory Profile from a national sample of children without disabilities. The American journal of occupational therapy : official publication of the American Occupational Therapy Association. 1997;51(7):490-5; discussion 6-9.

21. Ohl A, Butler C, Carney C, Jarmel E, Palmieri M, Pottheiser D, et al. Test-retest reliability of the sensory profile caregiver questionnaire. The American journal of occupational therapy : official publication of the American Occupational Therapy Association. 2012;66(4):483-7.
